# Post-Implant Phosphodiesterase-5 Inhibitors in Patients with Left Ventricular Assist Device: A Systematic Review and Meta-Analysis

**DOI:** 10.3390/jcm11205988

**Published:** 2022-10-11

**Authors:** Andrew Xanthopoulos, Dimitrios E. Magouliotis, Konstantinos Tryposkiadis, Prokopis-Andreas Zotos, Kyriakos Spiliopoulos, Thanos Athanasiou, Grigorios Giamouzis, John Skoularigis, Randall C. Starling, Filippos Triposkiadis

**Affiliations:** 1 Department of Cardiology, University Hospital of Larissa, 411 10 Larissa, Greece; 2 Department of Cardiothoracic Surgery, University of Thessaly, Biopolis Campus, 415 00 Larissa, Greece; 3 Independent Researcher, 156 69 Athens, Greece; 4 Department of Surgery and Cancer, Imperial College London, St. Mary’s Hospital, London W2 1NY, UK; 5 Kaufman Center for Heart Failure Treatment and Recovery, Heart, Vascular, and Thoracic Institute, Cleveland Clinic, Cleveland, OH 44195, USA

**Keywords:** left ventricular assist devices, phosphodiesterase-5 inhibitors, mortality, hemocompatibility, bleeding, thrombosis, meta-analysis

## Abstract

Background: Despite the improvement in left ventricular assist device (LVAD) technology and the advent of third-generation LVADs, hemocompatibility-related events remain a significant issue. Therefore, new pharmacological treatments are necessary to optimize patient management and to further reduce hemocompatibility-related events. The purpose of the present systematic review and meta-analysis was to summarize the existing data regarding the safety and efficacy of post-implant phosphodiesterase-5 inhibitors (PDE-5i) on hemocompatibility-related events. Methods: Among the 258 articles in Pubmed, Scopus, and CENTRAL that were retrieved (1990–2022), 15 studies were included in the qualitative synthesis, and 9 studies were included in the quantitative synthesis. The fixed-effects model was used because it is statistically sound for combining a very small number of studies. The primary endpoint of the study was all-cause mortality, whereas the secondary endpoints were ischemic stroke, pump thrombosis, and gastrointestinal bleeding. Results: Mortality was significantly lower in the PDE-5i group vs. the control group (OR: 0.92 [95% CI: 0.85, 0.98]; *p* = 0.02). The secondary endpoints ischemic stroke (OR: 0.87 [95% CI: 0.78, 0.98]; *p* = 0.02) and pump thrombosis (OR: 0.90 [95% CI: 0.82, 0.99]; *p* = 0.04) were also lower in the PDE-5i group. The incidence of gastrointestinal bleeding was significantly higher in patients with LVAD receiving PDE-5i (OR: 1.26 [95% CI: 1.11, 1.44]; *p* < 0.01). In the overall analysis, the heterogeneity of outcomes was low, except for pump thrombosis. Conclusions: The use of PDE-5i post-implant was associated with lower mortality and thrombotic events but with a higher risk of gastrointestinal bleeding.

## 1. Introduction

Over the last 20 years, left ventricular assist devices (LVADs) have emerged as an approved treatment option for patients with end-stage heart failure who are not eligible for heart transplantation [1]. The advent of second-generation pumps with continuous flow improved quality of life and survival compared to first-generation LVADs, but they were associated with a high rate of hemocompatibility-related events, such as stroke, pump thrombosis, and bleeding [2,3]. Third-generation (centrifugal-flow) LVADs reduced the hemocompatibility-related events, but the stroke incidence remained high [4,5,6,7]. For this reason, further improvements in LVAD technology, along with new pharmacological treatments, are necessary to optimize patient management and to further reduce hemocompatibility-related events [8].

Phosphodiesterase type 5 inhibitors (PDE-5i) are known to enhance nitric oxide–mediated vasodilation by inhibiting the degradation of cGMP and exhibit antiplatelet and antithrombotic effects, while their administration has been associated with favorable hemodynamic effects on right ventricular (RV) function [9,10]. Recent large observational studies examining the use of PDE-5i post-implant have reported conflicting results [11,12,13]. Therefore, the safety and efficacy of PDE-5i in patients with LVAD remain debatable. The purpose of the present systematic review and meta-analysis is to summarize the existing data in the literature regarding the effect of post-implant PDE-5i on hemocompatibility-related events, such as pump thrombosis, stroke, gastrointestinal bleeding, and mortality in patients with continuous-flow LVADs.

## 2. Materials and Methods

### 2.1. Search Strategy and Article Selection

The present study was conducted in accordance with a protocol agreed on by all authors and the Preferred Reporting Items for Systematic Reviews and Meta-Analyses 2020 [14]. A thorough literature search in Pubmed (Medline), Scopus (ELSEVIER), and the Cochrane Central Register of Controlled Studies (CENTRAL) (last search: 27 March 2022) was performed. The following terms were employed in every possible combination: “left-ventricular assist device”, “LVAD”, “sildenafil”, “tadalafil”, “vardenafil”, “phosphodiesterase-5 inhibitor”, and “PDE5i”. Inclusion criteria were (1) original reports with ≥10 patients, (2) written in English, (3) published from 1990 to 2022, (4) conducted on human subjects, and (5) reporting comparative outcomes of patients with LVAD receiving or not PDE-5i. Duplicate articles and articles without data on post-implant PDE-5i use were excluded. The reference lists of all included articles were also reviewed for additional studies. Two independent reviewers (AX and DEM) extracted data from the included studies. Any discrepancies between the investigators about the inclusion or exclusion of studies were discussed with the senior authors (FT and RCS) until a consensus was reached to include articles that best matched the criteria. The authors had personal equipoise with regard to the best intervention.

### 2.2. Definitions

Hemocompatibility refers to the consequences of either a harmonious relationship or an adverse interaction between the artificial pump interface and the activation or destruction of circulating blood elements [15]. In this regard, hemocompatibility-related events evaluated in the present meta-analysis included [15,16,17,18]: 

A. Gastrointestinal bleeding, defined as bleeding from the upper gastrointestinal tract, bleeding from the lower gastrointestinal tract, or bleeding of unknown origin but with guaiac-positive stools.

B. Ischemic stroke, defined as the sudden onset of neurologic signs or symptoms fitting a focal or multifocal vascular territory within the brain, spinal cord, or retina, with:

(1) Symptoms persisting for ≥24 h or until death, with pathology or neuroimaging evidence that demonstrates either: (a) central nervous system infarction in the corresponding vascular territory or (b) the absence of other apparent causes, even if no evidence of acute ischemia in the corresponding vascular territory is detected; 

or

(2) Symptoms lasting <24 h, with pathology or neuroimaging confirmation of central nervous system infarction in the corresponding vascular territory that was diagnosed by neurological experts using image studies.

C. Pump thrombosis, including (1) suspected device thrombosis, defined as a device-related malfunction, with signs and symptoms to include at least 1 of the 3 following criteria: (i) presence of major hemolysis, (ii) presence of heart failure not explained by structural heart disease, or (iii) abnormal pump parameters consistent with diminished pump output/pump efficiency/pump performance. Suspected device thrombosis is accompanied by 1 or more of the following events or interventions: death, stroke or transient ischemic attack, arterial non-central nervous system thromboembolism, de novo need for inotrope therapy, treatment with intravenous anti-coagulants, intravenous thrombolytics, or intravenous antiplatelet therapy, pump replacement, pump explantation with or without exchange, pump deactivation without pump removal, operation to repair or replace any internal component of the circulatory support system, or urgent transplantation listing; (2) confirmed device thrombosis, defined as a device-related malfunction in which thrombus was confirmed within the blood-contacting surfaces of device inflow cannula or outflow conduit or grafts.

### 2.3. Data Extraction

For each eligible study, data were extracted in relation to demographics (number of patients, sex, mean age, LVAD model, and type of PDE-5i), along with complications and mortality. The outcome related to mortality was the primary endpoint, while hemocompatibility-related complications were secondary endpoints. In addition, categorical outcomes were 2 × 2 tabulated, referring patients presenting the outcome and patients free of the outcome, separately for PDE-5i and control groups. Regarding continuous outcomes, we extracted the mean, the standard deviation, and the number of patients. If the standard deviation was not available, it was calculated using the available data. In cases where Propensity-Score-Matched (PSM) analysis was performed, the data from only PSM were included. The kappa coefficient test was applied as a measure of the level of agreement between the reviewers. 

### 2.4. Statistical Analysis 

Regarding the categorical outcomes, the Odds Ratio (OR) and 95% confidence interval (CI) were calculated based on the extracted data by means of a fixed-effects model (Mantel–Haenszel statistical method). OR < 1 denotes that the outcome was more frequent in the control group. Continuous outcomes were evaluated by means of the weighted mean difference (WMD) with its 95% CI using fixed-effects (Inverse Variance statistical method) models to calculate pooled effect estimates. In cases where WMD < 0, values in the control group were higher. Although a random-effects model provides a greater scope of inference, we chose the fixed-effects model because it is statistically sound for combining a very small number of studies [19]. Inter-study heterogeneity was assessed through Cochran’s Q statistic and by estimating I2 [20]. High heterogeneity renders the outcome less valid. A *p*-value of less than 0.05 was set as the threshold indicating a statistically significant result. We also performed a sensitivity analysis using the leave-one-out method to assess the quality of our findings. Where multiple studies analyzed the same population, only the larger study or the one with the longest follow-up was included in the qualitative and quantitative analyses. 

### 2.5. Quality and Publication Bias Assessment 

The Newcastle–Ottawa Quality Assessment Scale (NOS) [21] was used as an assessment tool to evaluate non-RCTs. The scale’s range varies from zero to nine stars, and studies with a score equal to or higher than five were considered to have the adequate methodological quality to be included. There were no RCTs in the literature to be included. Two reviewers (AX and DEM) rated the studies independently, and a final decision was reached by consensus. Visual inspection of funnel plot asymmetry was performed to address possible small-study effects.

## 3. Results

### 3.1. Search Strategy and Patient Demographics

**Figure 1:**
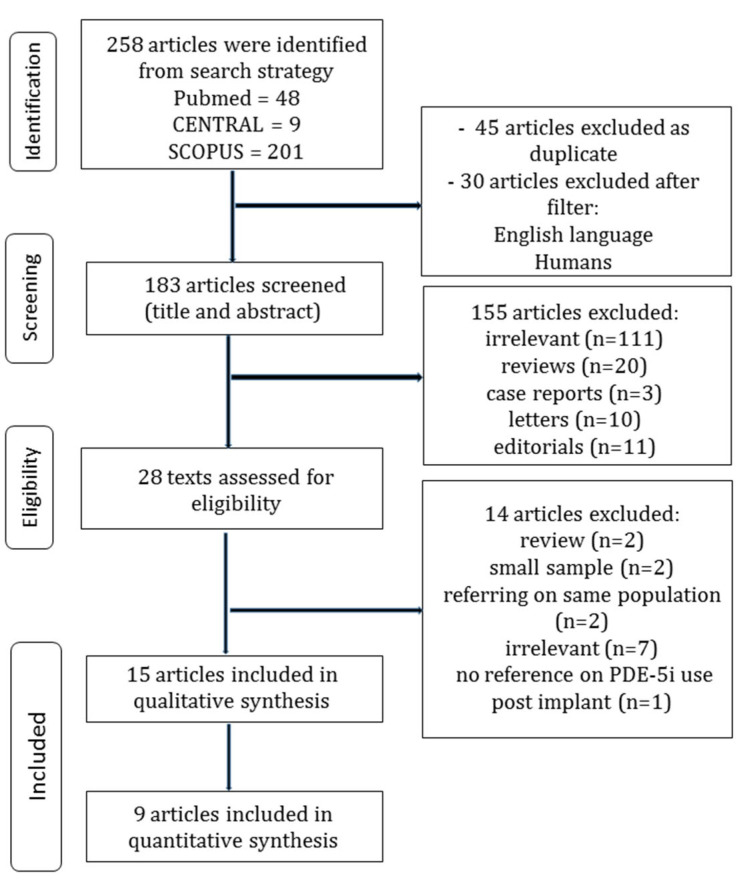
PDE5i vs. control treatment for patients with LVAD: flow diagram.

The flow diagram regarding the search strategy is shown in Figure 1, and the Prisma 2020 Checklist is provided in the Supplementary Table. The characteristics of the included studies are summarized in Table 1. Among the 258 articles in Pubmed, Scopus, and CENTRAL that were retrieved, 15 studies were included in the qualitative synthesis, and 9 studies were included in the quantitative synthesis [11,12,13,22,23,24,25,26,27]. The level of agreement between the two reviewers was “almost perfect” (kappa = 0.972; 95% CI: 0.917, 1.000). The study cohort was prospective in three studies [11,12,13] and retrospective in twelve studies [22,23,24,25,26,27,28,29,30,31,32,33]. The included studies were conducted in Australia [28], the United States of America [7,11,12,13,23,25,26,28,29,30,31], Lebanon [29], and Germany [22,27]. The total sample size was 25,599 patients. The baseline characteristics of the studies are provided in Table 1, and the pooled estimates are presented in Table 2. The Newcastle–Ottawa rating scale assessment for all studies is shown in Table 1. Patients in the PDE5i group were associated with younger age (Table 1).

The Newcastle–Ottawa Scale (NOS) was used to assess the quality of non-randomized studies. Every study was judged from three perspectives: selection, comparability, and the ascertainment of the exposure of the study groups. The highest-quality studies are awarded up to nine stars. Abbreviations: R = retrospective; P = prospective; ND = non-defined; CF-LVAD = continuous-flow LVAD; LVAD = left ventricular assist device; PDE5I = phosphodiesterase-5 inhibitors; HM2 = HeartMate II; HVAD = HeartWare VAD; HM3 = HeartMate 3; NOS = Newcastle–Ottawa Scale.

### 3.2. Primary Endpoint: Mortality

**Figure 2:**
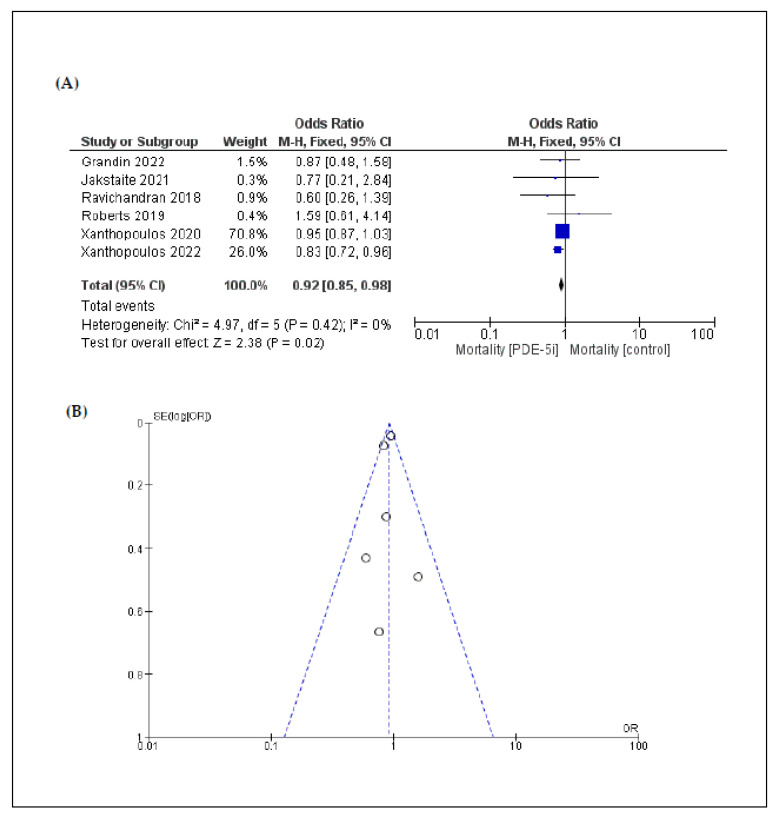
(**A**) Forest plot describing the differences in mortality between the 2 groups (PDE-5i vs. control); (**B**) funnel plot assessing the publication bias for mortality.

Mortality was assessed by a six-arm analysis. Mortality was significantly higher in patients in the control group (OR: 0.92 [95% CI: 0.85, 0.98]; *p* = 0.02) (Figure 2).

### 3.3. Secondary Endpoints: Complications

**Figure 3:**
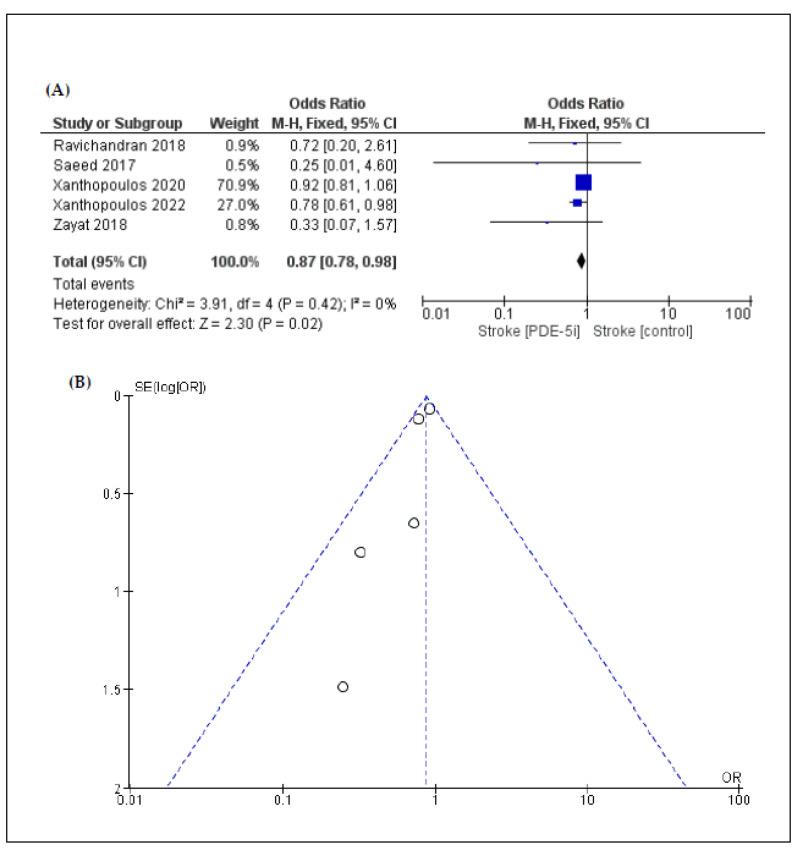
(**A**) Forest plot describing the differences in ischemic stroke between the 2 groups (PDE-5i vs. control); (**B**) funnel plot assessing the publication bias for ischemic stroke.

**Figure 4:**
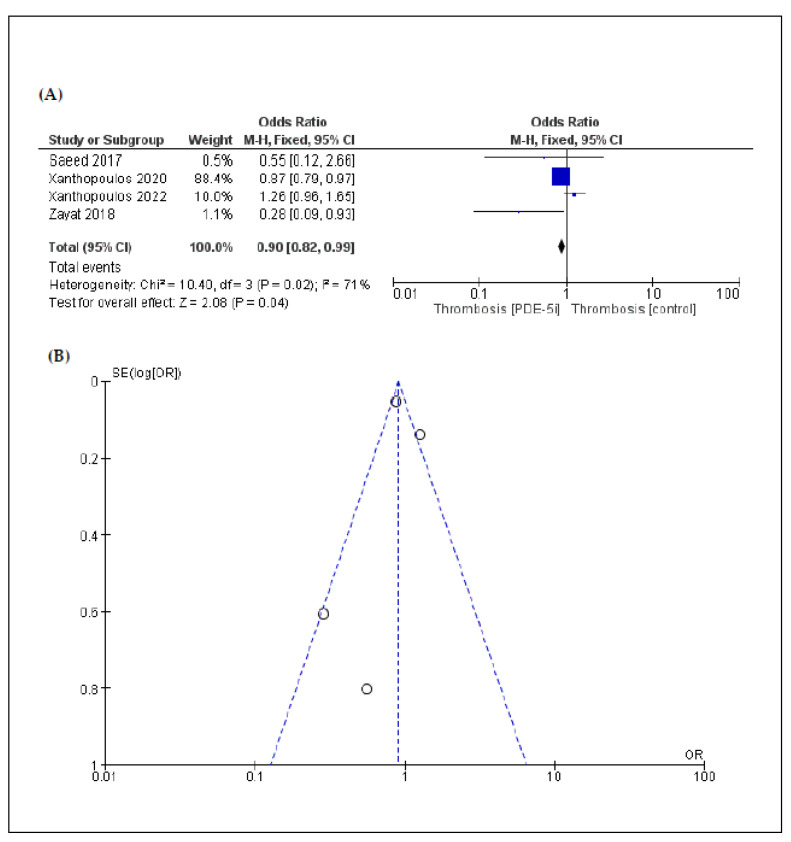
(**A**) Forest plot describing the differences in pump thrombosis between the 2 groups (PDE-5i vs. control); (**B**) funnel plot assessing the publication bias for pump thrombosis.

**Figure 5:**
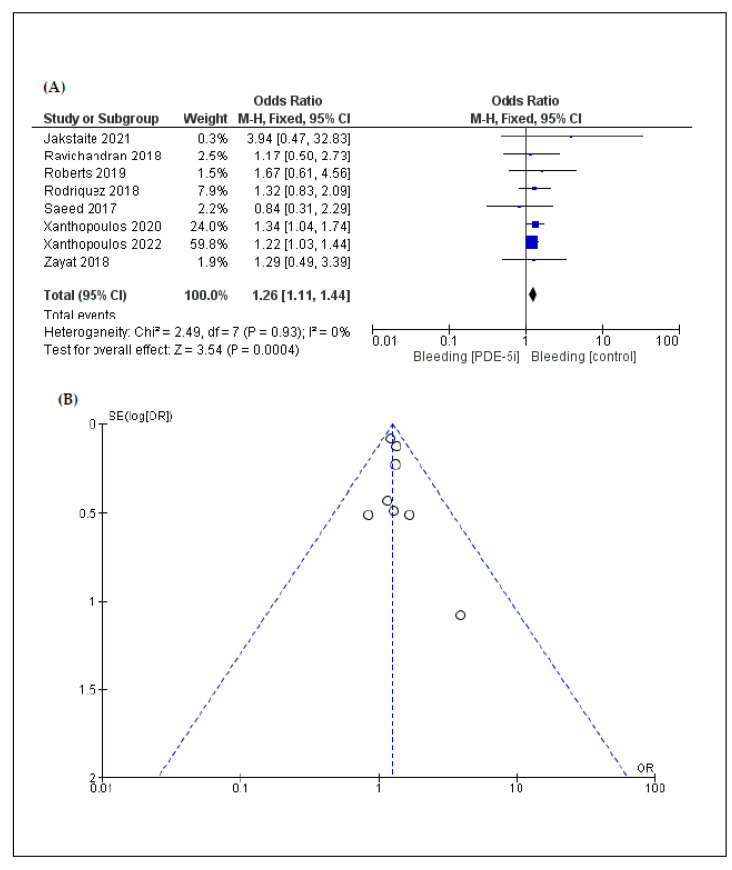
(**A**) Forest plot describing the differences in gastrointestinal bleeding between the 2 groups (PDE-5i vs. control); (**B**) funnel plot assessing the publication bias for gastrointestinal bleeding.

According to a five-arm analysis, the incidence of ischemic stroke (OR: 0.87 [95% CI: 0.78, 0.98]; *p* = 0.02) (Figure 3) was lower in the PDE-5i group compared to the control group. Similarly, the incidence of pump thrombosis (OR: 0.90 [95% CI: 0.82, 0.99]; *p* = 0.04) (Figure 4) was lower in the PDE-5i group. On the other hand, according to an eight-arm analysis, the incidence of gastrointestinal bleeding was significantly higher in patients with LVAD receiving PDE-5i (OR: 1.26 [95% CI: 1.11, 1.44]; *p* < 0.01) (Figure 5). 

### 3.4. Sensitivity Analysis

In the current meta-analysis, similar to the previous study by Kittipibul et al. [34], a conference abstract was included in the analysis of gastrointestinal bleeding [25]. Nevertheless, the results were similar after excluding this study from the analysis (Supplementary Figure S1).

No single study was found to affect our pooled outcomes regarding the primary and secondary endpoints or the level of heterogeneity when we performed the sensitivity analysis. The results of the meta-analysis regarding mortality and thrombotic events were reanalyzed following the exclusion of our two recent observational studies [11,12]. Mortality, although numerically lower in the PDE-5i group, was not significantly different between the two groups, whereas ischemic stroke and pump thrombosis endpoints did not reach statistical significance, but there was a trend favoring the use of PDE-5i (Supplementary Figures S2 and S3). It should be noted, however, that when our two studies were excluded from the meta-analysis, the number of patients was significantly lower (24,586 vs. 3585 for mortality; 21,350 vs. 349 for ischemic stroke; and 21,228 vs. 227 for pump thrombosis). Lastly, a sub-meta-analysis of the studies that reported the inclusion of patients with HeartMate 3 is listed in the Supplementary Material (Supplementary Figures S4 and S5).

### 3.5. Publication Bias

In the overall analysis, heterogeneity was low regarding the outcomes, except for pump thrombosis. Funnel plots that were produced to assess publication bias are shown in Figure 2, Figure 3, Figure 4 and Figure 5. Asymmetries that were found are mainly attributed to the small number of included studies and the bias regarding the selection of patients, the different LVAD models used, and the different protocols followed among different institutions, thus indicating that randomized control trials (RCTs) are necessary to fully eliminate publication bias.

## 4. Discussion

This systematic review and meta-analysis identified fifteen articles describing the impact of PDE-5i on mortality and hemocompatibility-related events (ischemic stroke, pump thrombosis, and gastrointestinal bleeding) in patients with implanted LVADs, published between 1990 and 2022. In this meta-analysis of observational studies, the administration of PDE-5i was associated with improved survival and fewer thrombotic events but with a higher risk of gastrointestinal bleeding.

Despite the improvement in LVAD technology, hemocompatibility-related events, especially ischemic stroke, remain an unresolved issue [6,7]. Therefore, new pharmacological approaches are needed, along with device progress, to optimize patient management. The implantation of an LVAD per se may exacerbate, via several mechanisms, pre-existing RV dysfunction in patients with advanced heart failure and lead to adverse outcomes, including death [35]. These observations have led to growing interest in the use of pulmonary vasodilators to treat pulmonary hypertension and prevent RV failure among LVAD-supported patients (see below) [35]. The 2013 International Society for Heart and Lung Transplantation guidelines state that PDE-5i “may be considered for management of RV dysfunction in the setting of pulmonary hypertension after mechanical circulatory support” (class of recommendation IIb, level of evidence C) [36]. Interestingly, data from large registries show that up to approximately 30% of LVAD patients receive off-label PDE-5i post-implant [11,12,13]. However, the safety and efficacy of this practice remain unclear [35].

Saeed et al. were the first to examine the relation between the administration of sildenafil and thrombotic events in a single-center study of 144 patients with implanted HeartMate II [26]. The authors reported an association between sildenafil administration and a lower risk of device thrombosis or ischemic stroke in patients with low-level hemolysis (defined as serum lactate dehydrogenase of 400 to 700 U/L). Consequently, few small studies have examined the association between PDE-5i administration and thrombotic events in LVAD patients, with conflicting results [24,27]. A propensity-matched analysis investigated the relationship between pre-implant PDE-5i administration and severe early right heart failure, defined as the composite of death from right heart failure within 30 days, the need for right ventricular assist device support within 30 days, or the use of inotropes beyond 14 days [37]. Higher rates of post-LVAD RV failure were observed in the pre-implant PDE-5i group, driven mainly by prolonged inotropic support. In addition, the cumulative incidence of major bleeding events at 1 month was higher in the PDE-5i group compared to controls (24.5% versus 17.9%) [37]. Interestingly, pre-implant PDE-5i use has not been associated with thrombotic risk reduction or increased survival [11,37]. Several mechanisms may explain adverse PDE-5i outcomes when administered before LVAD implantation, such as the development of hypotension and vasoplegia, a rise in pulmonary capillary wedge pressure from increased pulmonary blood flow directed towards a dilated, noncompliant left heart, and increased bleeding risk. In summary, the aforementioned analyses do not support the use of pre-implant PDE-5i therapy in LVAD candidates.

A significant association between post-LVAD-implant PDE-5i use and reduced thrombotic events, as well as improved survival, was reported in two observational studies [11,12]. The first included a cohort of 13,772 patients with continuous-flow LVADs participating in the Interagency Registry for Mechanically Assisted Circulatory Support (INTERMACS) between 2012 and 2017 [11]. Interestingly, the vast majority of those patients (~74%) were on a second-generation axial-flow LVAD, HeartMate II^®^. The primary endpoint (a composite of LVAD thrombosis and ischemic stroke) was significantly lower in the PDE-5i group compared with the no-PDE-5i group (hazard ratio (HR), 0.84; 95% CI, 0.77–0.91; *p* < 0.001) at 48 months. The secondary endpoint, all-cause mortality (HR, 0.86; 95% CI, 0.79–0.93; *p* < 0.001), was lower in the PDE-5i group versus the no-PDE-5i group during the study follow-up. The second study [12] investigated the association between post-implant PDE-5i use and outcomes in patients with implanted third-generation centrifugal-flow LVADs HeartMate 3 (*n* = 4628) and HVAD^®^ (*n* = 2601). The mean duration of follow-up was 11.94 ± 8.65 months. The primary composite endpoint (all-cause mortality, ischemic stroke, and pump thrombosis) was lower in the PDE-5i group (adjusted HR: 0.77; 95% CI: 0.69–0.86; *p* < 0.0001). In the present meta-analysis, the use of PDE-5i was associated with an 8% lower risk of all-cause mortality, a 13% lower risk of ischemic stroke, and a 10% lower risk of pump thrombosis. Interestingly, the observed heterogeneity regarding the outcomes was low (with the exception of pump thrombosis), making the possibility of publication bias unlikely.

PDE-5i exhibit several pleiotropic effects (cardiovascular and non-cardiovascular) that may explain the favorable outcomes observed in LVAD patients receiving the drug [38]. For example, PDE-5i have been shown to decrease pulmonary artery pressure, reverse RV remodeling, enhance RV–pulmonary artery coupling, and decrease diastolic leftward septal shift. PDE-5i also exhibit antiplatelet and antithrombotic actions. In this regard, not surprisingly, several studies have reported an association between PDE-5i and gastrointestinal bleeding [11,12,22,23,24,25,27,37]. This might be explained by the fact that PDE-5i exhibit antiplatelet and antithrombotic actions by potentiating the nitric oxide–mediated inhibition of platelet aggregation through the blockade of cGMP degradation [39,40,41]. Furthermore, despite the fact that in the studies by Xanthopoulos et al. [11,12], the frequencies of aspirin and antiplatelet agent (including aspirin) use, as well as the international normalized ratio (INR) values over time, were not significantly different between PDE-5i and non-PDE-5i groups, the possibility that the interaction of PDE-5i with coumadin or antiplatelets may lead to increased gastrointestinal bleeding, as reported in this meta-analysis, cannot be excluded. Nevertheless, the mechanisms of gastrointestinal bleeding in LVAD patients are multifactorial [42,43]. In this regard, RV failure has been shown to be a major risk factor for gastrointestinal bleeding in numerous previous studies [44,45]. Therefore, the association of PDE-5i with bleeding could be partially explained via “confounding by indication” [13]. In the present meta-analysis, patients on PDE-5i exhibited a 26% higher risk of gastrointestinal bleeding compared to the control group.

Grandin et al., utilizing a propensity-matched analysis from the INTERMACS Registry, reported similar rates of the primary endpoint, late right heart failure (HR, 1.14; 95% CI, 0.99–1.32; *p* = 0.07), and the secondary endpoint, mortality (HR, 0.99; 95% CI, 0.86–1.15; *p* = 0.93), in patients receiving early post-implant (i.e., 1 month) PDE-5i vs. the control group [13]. Nevertheless, the use of right heart failure as a study endpoint in LVAD patients is challenging [46]. First of all, the complex nature of right-sided heart failure, diverse pathways, and multispecialty involvement among distinct clinicians has led to the development of varying definitions over the last decade [47,48]. Secondly, those definitions often change with time, making the interpretation and comparison of right heart failure (as an outcome) among different studies difficult [13]. Thirdly, right heart dysfunction may be aggravated during exercise or periods of increased physiological needs [48]. Therefore, not surprisingly, there is currently no consensus on how to best measure RV function in clinical trials [49]. 

The timing of PDE-5i administration (pre- vs. post-LVAD implant) may explain the discrepancies observed between the study by Gulati et al. [37] and our studies [11,12]. In particular, when PDE-5i are given pre-LVAD implant [37], they may cause selective pulmonary vasodilation, resulting in an increase in RV output. The increased RV output cannot be accommodated by the dilated LV (exhaustion of the preload reserve), leading to the further deterioration of LV function and causing RV dysfunction due to ventricular interdependence (shift of the interventricular septum towards the RV) [50]. When treatment with PDE-5i is initiated post-LVAD implant [11,12], the increase in RV output induced by these agents may improve the filling of the supported LV and optimize cardiac output. The optimized hemodynamics resulting from the post-implant use of PDE-5i, along with the other pleiotropic effects of these agents, including their antiplatelet and antithrombotic effects, contribute to the reported reduction in the risk of ischemic strokes and all-cause mortality in LVAD patients [50]. 

In terms of the contradictory results observed in the studies by Xanthopoulos et al. [11,12] and Grandin et al. [13], these can be attributed to differences in study entry criteria. For example, the study by Grandin et al. [13] enrolled patients receiving a primary continuous-flow LVAD and surviving at least 30 days after discharge from the index hospitalization. In contrast, both of our studies [11,12] included patients who received PDE-5i early after LVAD implantation, and our findings (early separation of relevant curves, please see Supplementary Figures S6 and S7) strongly suggest that in the study of Grandin et al. [13], the early beneficial effect of PDE-5i post-LVAD implantation was probably missed.

Our study incorporates certain strengths compared to the previous meta-analysis by Kittipibul et al. [34]. First of all, we searched three databases compared with the two used in the previous meta-analysis. This led to a higher number of assessed articles included in the final analysis. Secondly, we used the fixed-effects model, given that it is more statistically sound for combining a small number of studies. Furthermore, the previous meta-analysis performed only three-arm analyses regarding hemocompatibility-related outcomes, while we performed five-arm analyses (for ischemic stroke), four-arm analyses (for pump thrombosis), and eight-arm analyses (for gastrointestinal bleeding), thus providing a significantly higher level of evidence. Lastly, the present meta-analysis included “hard endpoints” (i.e., mortality) that were not investigated in the meta-analysis by Kittipibul et al. [34].

### Strengths and Limitations

The limitations of the current meta-analysis reflect the limitations of the studies included. Most of the studies were retrospective, and no randomized controlled studies were identified. Furthermore, the differences among institutions regarding the selection criteria, the LVAD device, and the type of administered PDE-5i, along with postoperative management, should be acknowledged. Unfortunately, the studies with the largest number of patients [11,12,13] do not provide information about the type of PDE-5i used. Most of the studies refer to results obtained with LVADs that are no longer used and/or left the market due to evident thrombotic problems. Additionally, the difference between the two groups (PDE-5i vs. control group) regarding age represents another potential bias. Lastly, the weight of the studies by Xanthopoulos et al. [11,12] is relevant, although the heterogeneity is not significant.

On the other hand, the strengths of this study include (a) the clear data extraction protocol, (b) the well-specified inclusion/exclusion criteria, (c) a literature search performed in three databases, (d) the quality assessment of the included studies, (e) the detailed presentation of the results of data extraction and analysis, and (f) the performance of a sensitivity analysis.

## 5. Conclusions

The present meta-analysis of observational studies demonstrated an association between the post-implant use of PDE-5i and lower mortality as well as thrombotic events in LAVD patients. The risk of gastrointestinal bleeding was higher in the PDE-5i group. The current best available evidence favors the postoperative use of PDE-5i in LVAD patients; however, an RCT is needed to provide definite answers.

## Figures and Tables

**Table 1:** Characteristics of the studies that were finally included in the meta-analysis.

Study ID, Year	Country	Study Design	LVAD Model	Patients, *n*		Female, *n* (%)		Mean Age ± SD		Type of PDE5I	NOS
				PDE5i	Control	PDE5i	Control	PDE5i	Control		
Critoph et al., 2016 [28]	Australia	R	52% HVAD 11% HM2	4	61	ND	ND	ND	ND	Sildeanfil	5
Grandin et al., 2022 [13]	United States of America	P	CF-LVAD	1600	1600	334 (20.9)	345 (21.6)	56.3 ± 12.5	56.4 ± 12.7	ND	7
Hamdan et al., 2014 [29]	Lebanon	R	71% HM2 29% HVAD	8	6	3 (37.5)	1 (16.7)	34.3 ± 14.1	36.5 ± 16.5	Sildenafil	6
Hassanein et al., 2017 (abstract) [30]	United States of America	R	HM2/HVAD	59	14	10 (17)	4 (28.6)	57.0 ± 11.6	56.4 ± 11.3	Sildenafil/ Tadalafil	6
Jakstaite et al., 2021 [22]	Germany	R	90% HVAD 10% HM3	75	34	9 (12)	7 (21)	53 ± 13	57 ± 10	7 Sildenafil 68 Tadalafil	7
Mahankali et al., 2011 (abstract) [31]	United States of America	R	CF-LVAD	29	23	ND	ND	ND	ND	Sildenafil	5
Raina et al., 2012 (abstract) [32]	United States of America	R	CF-LVAD	80	16	ND	ND	ND	ND	Sildenafil	5
Ravichandran et al., 2018 [23]	United States of America	R	82% HM2 8% HVAD	53	69	35 (51)	19 (35)	55.5 ± 12.3	54.4 ± 13.5	Sildenafil	6
Roberts et al., 2019 [24]	United States of America	R	86% HM 2 14% HVAD	77	77	24 (31.2)	19 (24.7)	59.5 (51–64)	60.5 (53–66)	Sildenafil/ Tadalafil	7
Rodriguez et al., 2018 (abstract) [25]	United States of America	R	76.5% HM2 19.7% HVAD 3.8% HM3	119	199	ND	ND	ND	ND	Sildenafil/ Tadalafil	5
Saeed et al., 2017 [26]	United States of America	R	HM2	37	107	ND	ND	ND	ND	Sildenafil	6
Solomon et al., 2019 (abstract) [33]	United States of America	R	HM2	62	106	ND	ND	ND	ND	Sildenafil	5
Xanthopoulos et al., 2020 [11]	United States of America	P	CF-LVAD *	4950	8822	1063 (21.5)	1875 (21.3)	56 ± 13	58 ± 13	ND	8
Xanthopoulos et al., 2022 [12]	United States of America	P	64% HM3 36% HVAD	2173	5056	514 (23.7)	1142 (22.6)	56.2 ± 13.0	57.6 ± 13.0	ND	8
Zayat et al., 2018 [27]	Germany	R	HM2	56	27	ND	ND	ND	ND	Sildenafil	6

* 73.9% continuous-axial-flow LVAD and 26.1% continuous-centrifugal-flow LVAD.

**Table 2:** Summary of the overall analysis of categorical and continuous outcomes.

Categorical Outcomes	*n*	OR (95% CI)	*p*	Heterogeneity	
				*I^2^*	*p*
**PDE5I vs. Control**					
Female ratio	6	1.03 [0.97, 1.10]	0.34	74%	<0.01
Gastrointestinal bleeding	8	1.26 [1.11, 1.44]	<0.01	0%	0.93
Pump thrombosis	4	0.90 [0.82, 0.99]	0.04	71%	0.02
Ischemic stroke	5	0.87 [0.78, 0.98]	0.02	0%	0.42
Mortality	6	0.92 [0.85, 0.98]	0.02	0%	0.42
**PDE5I vs. Control**					
**Continuous Outcomes**	***n***	**WMD (95% CI)**	***p***	***I^2^***	***p***
Mean age	6	−1.44 [−1.74, −1.13]	<0.01	74%	<0.01

OR = Odds Ratio; WMD = weighted mean difference; CI = confidence interval; PDE5I = phosphodiesterase-5 inhibitor.

## Data Availability

The data that support the findings of this study are available from the corresponding author upon reasonable request.

## Supplementary Materials

The following supporting information can be downloaded at: https://www.mdpi.com/article/10.3390/jcm11205988/s1, Figure S1: (A) Forest plot describing the differences in gastrointestinal bleeding between the 2 groups (PDE-5i vs. control); (B) funnel plot assessing the publication bias for gastrointestinal bleeding. The conference abstract by Rodrigue et al. [25] was not included in the analysis; Figure S2: Forest plot describing the differences in mortality between the 2 groups (PDE-5i vs. control). The 2 large observational analyses by Xanthopoulos et al. [11,12] were not included in the analysis; Figure S3: Forest plot describing the differences between the 2 groups (PDE-5i vs. control): (A) in ischemic stroke and (B) in pump thrombosis. The 2 large observational analyses by Xanthopoulos et al. [11,12] were not included in the analysis; Figure S4: Sub-meta-analysis of studies with HeartMate 3 reporting all-cause mortality; Figure S5: Sub-meta-analysis of studies with HeartMate 3 reporting gastrointestinal bleeding; Figure S6: Cumulative incidence curves for all-cause mortality [11]; Figure S7: Cumulative incidence curves for all-cause mortality [12]; Prisma 2020 Checklist.

## References

[1] Gustafsson F., Rogers J.G. (2017). Left ventricular assist device therapy in advanced heart failure: Patient selection and outcomes. Eur. J. Heart Fail..

[2] Starling R.C., Moazami N., Silvestry S.C., Ewald G., Rogers J.G., Milano C.A., Rame J.E., Acker M.A., Blackstone E.H., Ehrlinger J. (2014). Unexpected abrupt increase in left ventricular assist device thrombosis. N. Engl. J. Med..

[3] Slaughter M.S., Rogers J.G., Milano C.A., Russell S.D., Conte J.V., Feldman D., Sun B., Tatooles A.J., Delgado R.M., Long J.W. (2009). Advanced heart failure treated with continuous-flow left ventricular assist device. N. Engl. J. Med..

[4] Colombo P.C., Mehra M.R., Goldstein D.J., Estep J.D., Salerno C., Jorde U.P., Cowger J., ClevelandJr J.C., Uriel N., Sayer G. (2019). Comprehensive Analysis of Stroke in the Long-Term Cohort of the MOMENTUM 3 Study. Circulation.

[5] Milano C.A., Rogers J.G., Tatooles A.J., Bhat G., Slaughter M.S., Birks E.J., Mokadam N.A., Mahr C., Miller J.S., Markham D.W. (2018). HVAD: The ENDURANCE Supplemental Trial. JACC Heart Fail..

[6] Mehra M.R., Uriel N., Naka Y., Cleveland J.C., Yuzefpolskaya M., Salerno C.T., Walsh M.N., Milano C.A., Patel C.B., Hutchins S.W. (2019). A Fully Magnetically Levitated Left Ventricular Assist Device—Final Report. N. Engl. J. Med..

[7] Rogers J.G., Pagani F.D., Tatooles A.J., Bhat G., Slaughter M.S., Birks E.J., Boyce S.W., Najjar S.S., Jeevanandam V., Anderson A.S. (2017). Intrapericardial Left Ventricular Assist Device for Advanced Heart Failure. N. Engl. J. Med..

[8] Hollis I.B., Doligalski C.T., Jennings D.J. (2021). Pharmacotherapy for durable left ventricular assist devices. Pharmacotherapy.

[9] Monzo L., Reichenbach A., Al-Hiti H., Borlaug B.A., Havlenova T., Solar N., Tupy M., Ters J., Kautzner J., Melenovsky V. (2021). Acute Unloading Effects of Sildenafil Enhance Right Ventricular-Pulmonary Artery Coupling in Heart Failure. J. Card. Fail..

[10] Hutchings D., Anderson S., Caldwell J.L., Trafford A.W. (2018). Phosphodiesterase-5 inhibitors and the heart: Compound cardioprotection?. Heart.

[11] Xanthopoulos A., Tryposkiadis K., Triposkiadis F., Fukamachi K., Soltesz E.G., Young J.B., Wolski K., Blackstone E.H., Starling R.C. (2020). Postimplant Phosphodiesterase Type 5 Inhibitors Use Is Associated With Lower Rates of Thrombotic Events After Left Ventricular Assist Device Implantation. J. Am. Heart Assoc..

[12] Xanthopoulos A., Wolski K., Wang Q., Blackstone E.H., Randhawa V.K., Soltesz E.G., Young J.B., Nissen S.E., Estep J.D., Triposkiadis F. (2022). Postimplant Phosphodiesterase-5 Inhibitor Use in Centrifugal Flow Left Ventricular Assist Devices. JACC Heart Fail..

[13] Grandin E.W., Gulati G., Nunez J.I., Kennedy K., Rame J.E., Atluri P., Pagani F.D., Kirklin J.K., Kormos R.L., Teuteberg J. (2022). Outcomes With Phosphodiesterase-5 Inhibitor Use After Left Ventricular Assist Device: A STS-INTERMACS Analysis. Circ. Heart Fail..

[14] Page M.J., McKenzie J.E., Bossuyt P.M., Boutron I., Hoffmann T.C., Mulrow C.D., Shamseer L., Tetzlaff J.M., Akl E.A., Brennan S.E. (2021). The PRISMA 2020 statement: An updated guideline for reporting systematic reviews. BMJ.

[15] Mehra M.R. (2019). The burden of haemocompatibility with left ventricular assist systems: A complex weave. Eur. Heart J..

[16] Uriel N., Colombo P.C., Cleveland J.C., Long J.W., Salerno C., Goldstein D.J., Patel C.B., Ewald G.A., Tatooles A.J., Silvestry S.C. (2017). Hemocompatibility-Related Outcomes in the MOMENTUM 3 Trial at 6 Months: A Randomized Controlled Study of a Fully Magnetically Levitated Pump in Advanced Heart Failure. Circulation.

[17] Mehra M.R., Crandall D.L., Gustafsson F., Jorde U.P., Katz J.N., Netuka I., Uriel N., Connors J.M., Sood P., Heatley G. (2021). Aspirin and left ventricular assist devices: Rationale and design for the international randomized, placebo-controlled, non-inferiority ARIES HM3 trial. Eur. J. Heart Fail..

[18] Kormos R.L., Antonides C.F., Goldstein D.J., Cowger J.A., Starling R.C., Kirklin J.K., Rame J.E., Rosenthal D., Mooney M.L., Caliskan K. (2020). Updated definitions of adverse events for trials and registries of mechanical circulatory support: A consensus statement of the mechanical circulatory support academic research consortium. J. Heart Lung Transplant..

[19] Borenstein M., Hedges L.V., Higgins J.P.T., Rothstein H.R. (2010). A basic introduction to fixed-effect and random-effects models for meta-analysis. Res. Synth. Methods.

[20] Higgins J.P.T., Green S. (2011). Cochrane Handbook for Systematic Reviews of Interventions Version 5.1.0. Updated March 2011. The Cochrane Collaboration. www.cochrane-handbook.org.

[21] Stang A. (2010). Critical evaluation of the Newcastle-Ottawa scale for the assessment of the quality of nonrandomized studies in meta-analyses. Eur. J. Epidemiol..

[22] Jakstaite A., Luedike P., Schmack B., Pizanis N., Riebisch M., Weymann A., Kamler M., Ruhparwar A., Rassaf T., Papathanasiou M. (2021). Increased bleeding risk with phosphodiesterase-5 inhibitors after left ventricular assist device implantation. ESC Heart Fail..

[23] Ravichandran A.K., LaRue S.J., Novak E., Joseph S.A., Schilling J.D. (2018). Sildenafil in Left Ventricular Assist Device Is Safe and Well-Tolerated. ASAIO J..

[24] Roberts K.L., Shuster J.E., Britt N.S., Balsara K.R., Graetz T.J., Helwani M., Itoh A., Tellor B.R. (2019). Evaluation of Clinical Outcomes with Phosphodiesterase-5 Inhibitor Therapy for Right Ventricular Dysfunction After Left Ventricular Assist Device Implantation. ASAIO J..

[25] Rodriguez L.C., Lawrecki T.M., Graney N., Siemeck R., Bhat G., Gavrilos G. (2018). Evaluation of gastrointestinal bleeds in left ventricular assist device patients receiving phosphodiesterase 5 inhibitors. ASAIO J..

[26] Saeed O., Rangasamy S., Selevany I., Madan S., Fertel J., Eisenberg R., Aljoudi M., Patel S.R., Shin J., Sims D.B. (2017). Sildenafil Is Associated With Reduced Device Thrombosis and Ischemic Stroke Despite Low-Level Hemolysis on Heart Mate II Support. Circ. Heart Fail..

[27] Zayat R., Ahmad U., Stoppe C., Khattab M.A., Arab F., Moza A., Tewarie L., Goetzenich A., Autschbach R., Schnoering H. (2018). Sildenafil Reduces the Risk of Thromboembolic Events in HeartMate II Patients with Low-Level Hemolysis and Significantly Improves the Pulmonary Circulation. Int. Heart J..

[28] Critoph C., Green G., Hayes H., Baumwol J., Lam K., Larbalestier R., Chih S. (2016). Clinical Outcomes of Patients Treated With Pulmonary Vasodilators Early and in High Dose After Left Ventricular Assist Device Implantation. Artif. Organs.

[29] Hamdan R., Mansour H., Nassar P., Saab M. (2014). Prevention of right heart failure after left ventricular assist device implantation by phosphodiesterase 5 inhibitor. Artif. Organs.

[30] Hassanein M.T., Sheikh F.H., Boyce S.W., Mohammed S.F., Hofmeyer M., Majure D.T. (2017). Phosphodiesterase type 5 inhibitor use does not affect heart failure hospitalizations in patients with left ventricular assist devices. J. Card. Fail..

[31] Sridhar A.M., Mukherjee V., Patel D., Ruiz G., Chawla R., Boyce S., Najjar S. (2011). Single center experience in the use of sildenafil for right ventricular dysfunction after LVAD implantation. J. Heart Lung Transplant..

[32] Raina A., Kanwar M., Sokos G., Moraca R., Bailey S., Benza R.L., Murali S. (2012). An aggressive, targeted perioperative management strategy results in low rates of postoperative right ventricular failure after left ventricular assist device implantation. J. Card. Fail..

[33] Solomon R., Obi C., Sharma S., Smith Z., Gheewala N., Cabrera R., Lanfear D., Jennings D., Long T. (2019). Hemodynamic effects of sildenafil on right heart function in left ventricular devices. J. Am. Coll. Cardiol..

[34] Kittipibul V., Blumer V., Angsubhakorn N., Hernandez G.A., Chaparro S., Tedford R.J., Agarwal R. (2021). Phosphodiesterase-5 Inhibitors and Outcomes during Left Ventricular Assist Device Support: A Systematic Review and Meta-Analysis. J. Card. Fail..

[35] Sparrow C.T., LaRue S.J., Schilling J.D. (2018). Intersection of Pulmonary Hypertension and Right Ventricular Dysfunction in Patients on Left Ventricular Assist Device Support: Is There a Role for Pulmonary Vasodilators?. Circ. Heart Fail..

[36] Feldman D., Pamboukian S.V., Teuteberg J.J., Birks E., Lietz K., Moore S.A., Morgan J.A., Arabia F., Bauman M.E., Buchholz H.W. (2013). The 2013 International Society for Heart and Lung Transplantation Guidelines for mechanical circulatory support: Executive summary. J. Heart Lung Transplant..

[37] Gulati G., Grandin E.W., Kennedy K., Cabezas F., DeNofrio D.D., Kociol R., Rame J.E., Pagani F.D., Kirklin J.K., Kormos R.L. (2019). Preimplant Phosphodiesterase-5 Inhibitor Use Is Associated With Higher Rates of Severe Early Right Heart Failure After Left Ventricular Assist Device Implantation. Circ. Heart Fail..

[38] Jorde U.P., Saeed O. (2022). Association of Improved Outcomes and Phosphodiesterase-5 Inhibition during Contemporary LVAD Support: End of the Beginning?. JACC Heart Fail..

[39] Halcox J.P., Nour K.R., Zalos G., Mincemoyer R., Waclawiw M.A., Rivera C.E., Willie G., Ellahham S., Quyyumi A.A. (2002). The effect of sildenafil on human vascular function, platelet activation, and myocardial ischemia. J. Am. Coll. Cardiol..

[40] Gudmundsdóttir I.J., McRobbie S.J., Robinson S.D., Newby D.E., Megson I.L. (2005). Sildenafil potentiates nitric oxide mediated inhibition of human platelet aggregation. Biochem. Biophys. Res. Commun..

[41] Gulati G., Kiernan M.S. (2020). Phosphodiesterase-5 Inhibitor Therapy for Left Ventricular Assist Device Patients: More Data, More Questions. J. Am. Heart Assoc..

[42] Imamura T., Kinugawa K., Uriel N. (2018). Therapeutic Strategy for Gastrointestinal Bleeding in Patients with Left Ventricular Assist Device. Circ. J..

[43] Klaeske K., Dieterlen M., Eifert S., Scholz U., Garbade J., Jawad K., Sieg F., Borger M.A., Meyer A.L. (2021). Device-induced platelet dysfunction in patients after left ventricular assist device implantation. J. Thromb. Haemost..

[44] Sparrow C.T., Nassif M.E., Raymer D.S., Novak E., LaRue S.J., Schilling J.D. (2015). Pre-Operative Right Ventricular Dysfunction Is Associated with Gastrointestinal Bleeding in Patients Supported With Continuous-Flow Left Ventricular Assist Devices. JACC Heart Fail..

[45] Yin M.Y., Ruckel S., Kfoury A.G., McKellar S.H., Taleb I., Gilbert E.M., Nativi-Nicolau J., Stehlik J., Reid B.B., Koliopoulou A. (2018). Novel Model to Predict Gastrointestinal Bleeding During Left Ventricular Assist Device Support. Circ. Heart Fail..

[46] Raina A., Patarroyo-Aponte M. (2018). Prevention and Treatment of Right Ventricular Failure During Left Ventricular Assist Device Therapy. Crit. Care Clin..

[47] Mehra M.R., Park M.H., Landzberg M.J., Lala A., Waxman A.B. (2014). International Right Heart Failure Foundation Scientific Working G. Right heart failure: Toward a common language. J. Heart Lung Transplant..

[48] Amsallem M., Mercier O., Kobayashi Y., Moneghetti K., Haddad F. (2018). Forgotten No More: A Focused Update on the Right Ventricle in Cardiovascular Disease. JACC Heart Fail..

[49] Lahm T., Douglas I.S., Archer S.L., Bogaard H.J., Chesler N., Haddad F., Hemnes A.R., Kawut S.M., Kline J.A., Kolb T.M. (2018). Assessment of Right Ventricular Function in the Research Setting: Knowledge Gaps and Pathways Forward. An Official American Thoracic Society Research Statement. Am. J. Respir. Crit. Care Med..

[50] Triposkiadis F., Xanthopoulos A., Skoularigis J., Starling R.C. (2022). Therapeutic augmentation of NO-sGC-cGMP signalling: Lessons learned from pulmonary arterial hypertension and heart failure. Heart Fail. Rev..

